# Innovative deep learning classifiers for breast cancer detection through hybrid feature extraction techniques

**DOI:** 10.1038/s41598-025-06669-4

**Published:** 2025-07-01

**Authors:** S. Vijayalakshmi, Binay Kumar Pandey, Digvijay Pandey, Mesfin Esayas Lelisho

**Affiliations:** 1 Department of Electronics and Communication Engineering, Sona College of Technology, Salem, 636005 Tamilnadu India; 2 Department of Information Technology, College of Technology, Govind Ballabh Pant University of Agriculture and Technology, Pantnagar, Uttarakhand India; 3 Department of Technical Education Uttar Pradesh, Kanpur, India; 4 Department of Statistics, College of Natural and Computational Science, Mizan-Tepi University, Tepi, Ethiopia

**Keywords:** Mammogram images, Deep learning, Grey level co-occurrence, Grey level difference matrix, Gray-level run-length matrix, Nakagami distribution parameter, Machine learning, Data integration, Data processing, Image processing, Cancer

## Abstract

Breast cancer remains a major cause of mortality among women, where early and accurate detection is critical to improving survival rates. This study presents a hybrid classification approach for mammogram analysis by combining handcrafted statistical features and deep learning techniques. The methodology involves preprocessing with the Shearlet Transform, segmentation using Improved Otsu thresholding and Canny edge detection, followed by feature extraction through Gray Level Co-occurrence Matrix (GLCM), Gray Level Run Length Matrix (GLRLM), and 1st-order statistical descriptors. These features are input into a 2D BiLSTM-CNN model designed to learn spatial and sequential patterns in mammogram images. Evaluated on the MIAS dataset, the proposed method achieved 97.14% accuracy, outperforming several benchmark models. The results indicate that this hybrid strategy offers improvements in classification performance and may assist radiologists in more effective breast cancer screening.

## Introduction

**Fig. 1:**
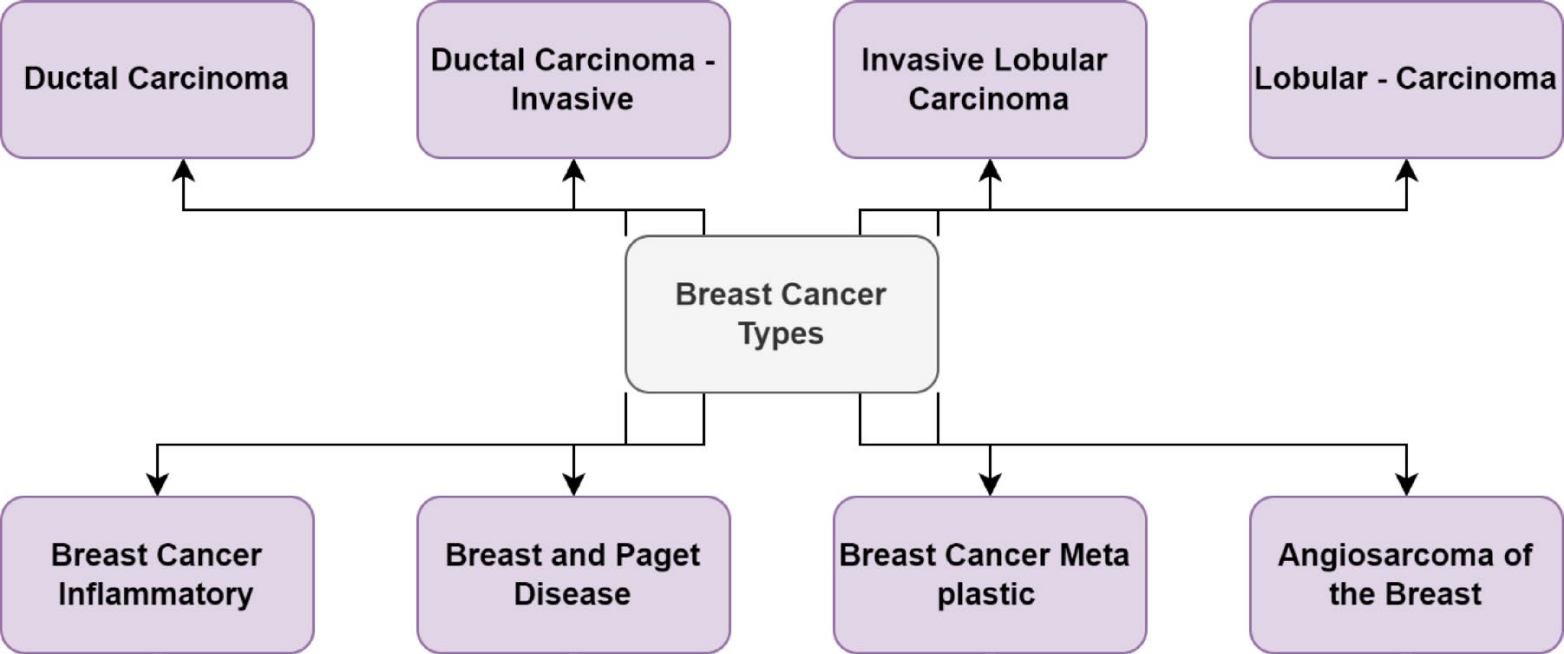
Illustration of different types of breast cancer based on shape, margin, and density as typically seen in mammogram imaging.

Breast cancer is a malignant disease that originates in the breast tissue, typically affecting the lobules and ducts responsible for milk production. It remains the most frequently diagnosed cancer and a leading cause of cancer-related mortality among women globally. According to recent global cancer statistics, early detection and timely diagnosis are critical to reducing mortality and improving prognosis. However, accurate detection remains a significant clinical challenge due to various factors, including image noise, subtle lesion features in early-stage cancers, and limitations in current imaging and diagnostic systems. Different forms of breast tumors are depicted in Fig. 1.

Conventional diagnostic imaging techniques such as mammography, ultrasound imaging, magnetic resonance imaging (MRI), and positron emission mammography (PEM)**—**play a crucial role in screening and diagnosing breast cancer. Among these, mammography has emerged as the primary tool for mass screening due to its accessibility, cost-effectiveness, and high spatial resolution. Nevertheless, mammograms are often prone to poor contrast, dense tissue overlap, and noise, which significantly hinder the radiologist’s ability to identify microcalcifications and masses accurately^1–3^. The manual interpretation of mammograms is further limited by inter-reader variability and the difficulty of detecting subtle changes, especially in dense glandular tissues. To address these challenges, researchers have explored various computer-aided diagnosis (CAD) systems that utilize machine learning (ML) and deep learning (DL) models to enhance diagnostic performance. Traditional ML models rely on handcrafted features such as intensity, shape, and texture descriptors derived from the regions of interest. Although these techniques offer some improvements in detection accuracy, they are constrained by their dependency on prior feature engineering knowledge and may not generalize well to complex and heterogeneous tissue patterns. Recently, deep learning models, particularly Convolutional Neural Networks (CNNs), have demonstrated remarkable performance in image classification tasks by learning high-level representations directly from image data^4,5^. Despite their potential, standalone CNN models are not without limitations in medical imaging contexts. CNNs excel in extracting spatial features but often fail to capture the temporal or sequential correlations in the pixel-wise feature evolution across regions, especially when applied to mammograms with intricate texture transitions. Furthermore, deep CNNs are computationally expensive and prone to overfitting when trained on small datasets like MIAS, leading to reduced generalizability. Several prior studies have explored CNN-based or hybrid DL models for breast cancer detection^6–10^, yet challenges remain in achieving optimal sensitivity and specificity while keeping the model efficient and clinically feasible. Another critical issue lies in the feature representation of mammogram images. Although deep learning models can automatically learn discriminative features, the absence of low-level statistical texture descriptors may lead to the loss of subtle cues vital for early detection. Handcrafted texture features such as Gray Level Co-occurrence Matrix (GLCM), Gray Level Run Length Matrix (GLRLM), and 1st-order statistical features have been shown to capture essential spatial dependencies and intensity distributions, which are often overlooked by end-to-end models. However, in most existing literature, these features are either used in isolation or fused with shallow classifiers, limiting their potential for complex pattern learning.

## Research gap

Given these limitations, there is a clear research gap in the development of a unified framework that can effectively combine the interpretability and robustness of handcrafted texture descriptors with the learning capacity of deep neural networks, particularly those capable of modeling sequential or temporal relationships within the data. Moreover, many current approaches rely solely on standard preprocessing techniques and lack a comprehensive enhancement pipeline that improves image quality before segmentation and classification. Additionally, few studies have explored the use of Bi-directional Long Short-Term Memory (BiLSTM) networks in conjunction with CNNs for mammogram-based breast cancer detection—despite BiLSTMs being well-suited to retain context across sequential features, which can enhance classification performance when combined with CNN-derived spatial features. Despite advancements in deep learning and image processing, existing breast cancer diagnostic tools often struggle with image noise, subtle lesion patterns, and inconsistent texture representation, leading to suboptimal classification results. This study therefore investigates the following research question

Can the integration of hybrid feature extraction techniques (GLCM, GLRLM, and 1st-order statistics) with a novel 2D BiLSTM-CNN classifier significantly enhance the accuracy, sensitivity, and specificity of breast cancer classification from mammogram images when compared to existing deep learning models?

### Motivation

- The early detection of breast cancer is crucial, yet current methods often fall short due to image noise and subtle early-stage signs.
- Enhancing image quality and feature extraction methods can significantly aid radiologists in making accurate diagnoses.
- Utilizing deep learning models and hybrid feature extraction techniques can improve the sensitivity and specificity of breast cancer detection.

### Contributions of research work

- Novel hybrid feature extraction: We introduce a hybrid feature extraction technique that combines the Gray Level Co-occurrence Matrix (GLCM), Gray Level Run Length Matrix (GLRLM), and 1st order statistical features to enhance the detection of breast cancer from mammogram images.
- Enhanced image preprocessing: Our methodology employs the Shearlet Transform for image enhancement, followed by Improved Otsu thresholding and Canny Edge Detection for effective segmentation of mammogram images.
- 2D BiLSTM-CNN classifier: We propose a 2D BiLSTM-CNN deep learning classifier that integrates the extracted features to classify normal and abnormal breast tissues with high accuracy.
- Comprehensive evaluation: The proposed method is rigorously evaluated on the MIAS dataset, demonstrating superior performance in terms of accuracy, sensitivity, and specificity compared to existing techniques.

This work aims to provide a robust tool for the early and accurate detection of breast cancer, thereby potentially reducing the mortality rate associated with this disease.

### Novelty of the work

The novelty of our proposed work lies in the innovative integration of hybrid feature extraction techniques and a unique deep learning architecture to enhance breast cancer detection. Unlike existing methods that typically rely on singular feature extraction techniques, we combine the Gray Level Co-occurrence Matrix (GLCM), Gray Level Run Length Matrix (GLRLM), and 1st order statistical features to capture a comprehensive set of textural and statistical properties from mammogram images. Furthermore, we introduce an advanced image preprocessing pipeline using the Shearlet Transform, Improved Otsu thresholding, and Canny Edge Detection to significantly improve image quality and segmentation. The core of our contribution is the 2D BiLSTM-CNN classifier, which uniquely integrates spatial features extracted by CNN with the sequential dependencies captured by BiLSTM networks, resulting in superior classification performance. This comprehensive approach is rigorously validated on the MIAS dataset. Results demonstrate significant improvements in accuracy, sensitivity, and specificity over existing methods, making it a robust tool for early breast cancer detection.

## Materials and methods

**Fig. 2:**
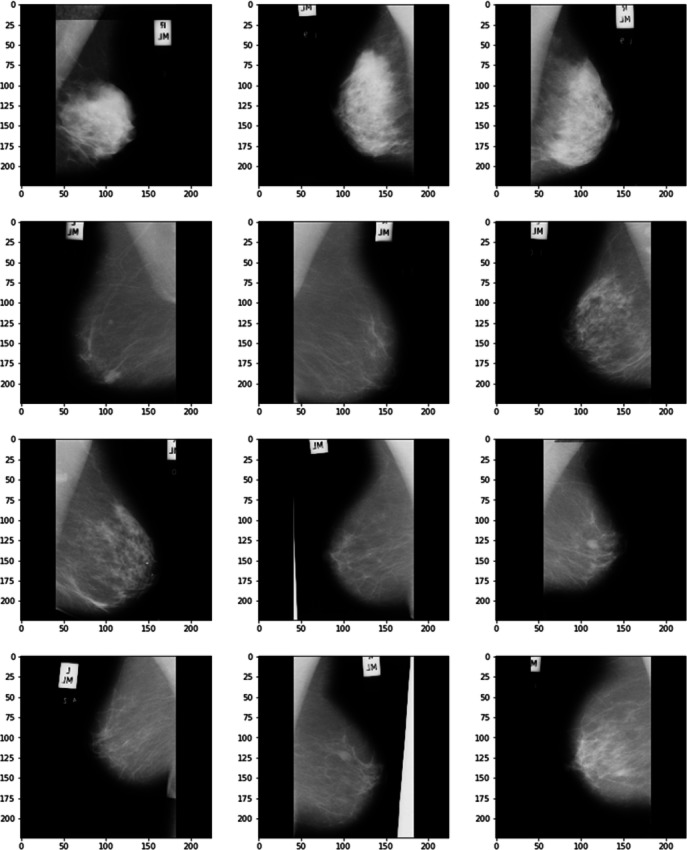
Example of a cranio-caudal (CC) mammographic view highlighting anatomical landmarks such as the pectoral muscle and retro-mammary space.

Cranio-Caudal projection is the most commonly used method for measuring the breast region in mammography^11^. When the breast is shifted forward, the pectoral muscle on the back edge of the breast region can be properly projected. This is an extremely uncommon outcome. Figure 2 depicts cranio-caudal mammographic scans. It is significant to note that the best CC placement is in the retromammary space, which is where the pectoral muscle visible on the back’s posterior border may be seen clearly. The areolar tissue in the retromammary area serves as a divider between the breast and the pectoral muscles on the chest. Due to its location far from the nervous system, this area represents the implantation of the breast and would support the breast region^1^. To construct a multi-view conspiracy, a single-view detection framework is used in this manner. Images from MLO and CC mammograms are generated independently using a single-view detection system.

It is significant to note that the best CC placement is in the retromammary space, which is where the pectoral muscle visible on the back’s posterior border may be seen clearly. The areolar tissue in the retromammary area serves as a divider between the breast and the pectoral muscles on the chest. Due to its location far from the nervous system, this area represents the implantation of the breast and would support the breast region^1^. To construct a multi-view conspiracy, a single-view detection framework is used in this manner. Images from MLO and CC mammograms are generated independently using a single-view detection system. This method includes several steps, such as dividing and pre-preparing, identifying questionable areas, and dividing the image area prior to single-view characterization.

### Various techniques for breast screening methods

Breast cancer screening is the process of examining a woman’s breast to see if she has cancer before any symptoms show up. The primary goal of breast cancer screening is to catch the disease in its earliest stages^2^. Breast cancer mortality rates can be reduced by early detection. Mammography, ultrasound, magnetic resonance imaging, and other techniques are used in the medical field to screen breast cancer.

#### Breast examination

Breast self-examination (BSE) and clinical breast examination (CBE) are two types of physical exams performed to check for lumps, edoema, or discharge from the nipple in the breast (CBE). Using a BSE, women can check their own breasts on a regular basis for physical changes. The physical examination of the breast is performed by health care experts in CBE. As one of the most basic ways to detect early-stage tumors, breast examinations are highly recommended. However, non-palpable lesions and their malignancy are not detected.

#### Mammography

Digital Mammography is a cutting-edge tool for the early detection of breast cancer. Specialized mammography uses digital receptors and computers instead of x-ray film to aid in the examination of breast tissue for cancer and to allow radiologists to clearly observe the results. Mammography is the standard for early breast cancer identification, with a high cure rate for women who undergo the procedure. Mammograms can detect abnormalities in the breast that may be cancerous before they cause any symptoms^3^. Early identification improves survival and treatment options, according to a slew of research.

Mammography has several advantages, including its low cost of implementation for a large number of people. Because radiologists review an average of more than a hundred films per day, it is difficult to maintain consistency and accuracy in diagnosis. Computer assisted diagnostic methods therefore hold the most promise for enhancing the initial diagnosis of breast cancer and reducing the associated mortality rates. In most cases, the contrast and noise in mammogram pictures are modest. Microcalcifications and masses are difficult to detect as a result. The presence of abnormally high levels of calcium in the breast is an indication of early breast cancer and should not be ignored. The pictures in a mammogram must be of the highest quality in order to correctly interpret this deposition.

#### Ultrasound

Anatomical structures, tissue characterization, and blood flow measures can all be obtained using ultrasound imaging (UI). The pulses transmitted by a typical ultrasonic machine use a transducer with a piezoelectric crystal. Raw data for images is created by converting output echoes into a voltage signal. UI pictures of the breast are sectional images; hence they require experienced clinicians for acquisition and interpretation. Breast UI scans have the unfortunate side effect of necessitating an excessive number of images to properly interpret the results^4^. As a result, performance degrades, noise levels rise, and boundary details become obscured. UI is a more difficult imaging modality when compared to others.

#### Magnetic resonance imaging

As the name implies, magnetic resonance imaging makes use of nuclear magnetic resonance to achieve its results (MRI). Atoms and protons’ quantum properties are mostly responsible for this. In medical imaging, it’s a multidimensional modality. As an alternative to X-rays, radio waves and magnetization can be utilized to produce highly detailed cross-sectional images^5^. Gadolinium DTPA contrast material is injected into an arm vein before or during an MRI scan to better see the breast features. In addition to the difficulty of normalizing inter- and interscan images, patient movement also causes problems. It provides up-to-the-minute data about angiogenesis. MRI scan’s main disadvantages are difficulty in detecting microcalcifications, inaccuracies in abnormality identification, and a higher price. Besides that, established procedures of analysis and clarification are required.

#### Positron emission mammography

Medical professionals utilise Positron Emission Mammography (PEM) to assess the severity of certain disorders. Uses gamma radiation and radioactive tracer to diagnose abnormalities in the breast tissue. Poisson mammography uses PEM has a 72–94% specificity range and a 1.0–2.4 mm spatial resolution. As the radiopharmaceuticals enter the body, they are stored and gradually degrade in radioactivity. Healthy and malignant cells are both affected by it^12^. In comparison to other approaches, PEM is time-consuming and luxurious. A variety of medical imaging technologies are now available through Biomedical Technology. However, each method has its own advantages and disadvantages. When it comes to breast cancer screening, accuracy comes at a price. The cost of screening is prohibitive for most people in developing nations.

#### Molecular imaging

It is also known as functional imaging since it offers information on the metabolic activity of a certain organ or tissue. By discharging nuclear energy and emitting photons such as gamma photons and/or certain elements, such as alpha and positron elements, radioactivity is used to turn an imbalanced nucleus into an evenly balanced nucleus. Single photon emission computed tomography is another name for gamma ray imaging that uses radioisotopes injected into the bodily tissue as a source of emission of gamma-ray radiation. A computer reconstructs images from the raw data collected by the detectors, which capture the released gamma rays. The attenuation and dispersion difficulties make it difficult to uncover structural details, although it is useful for spotting metastases^13^. A Positron Emission Tomography (PeT) scan is used to detect biochemical movements and injected radioactive materials’ physiological responses in tissue (PET). It can also be used to detect tissue glucose absorption and blood circulation, and two indicators of cancer spread. Because of the high cost, low resolution, and noise in the reconstructed images, this method is not recommended.

#### Digital tomosynthesis

In Digital Tomosynthesis, several photos of a trampled breast are taken in a short period of time at a stationary trampled breast. 3D imaging is what it is. As a result of this screening procedure, the abnormal component is more easily identified when surrounding areas are obscured, and it also provides increased accuracy, reduced breast compression, and 3D lesion localization, as well as enhancing imaging in three dimensions (three-dimensional enhancement). This method has a few drawbacks, including a longer acquisition time, a higher radiation dosage, and a longer processing time.

## Proposed work

### Overview of the framework

The objective of the proposed framework is to improve the accuracy and robustness of breast cancer classification by combining advanced image preprocessing techniques, hybrid feature extraction strategies, and a novel 2D BiLSTM-CNN classifier. The complete pipeline consists of four major stages: image acquisition and preprocessing, region of interest segmentation, hybrid feature extraction, and classification using a deep neural model. This section details each of these stages comprehensively to facilitate reproducibility and technical clarity.

**Fig. 3:**
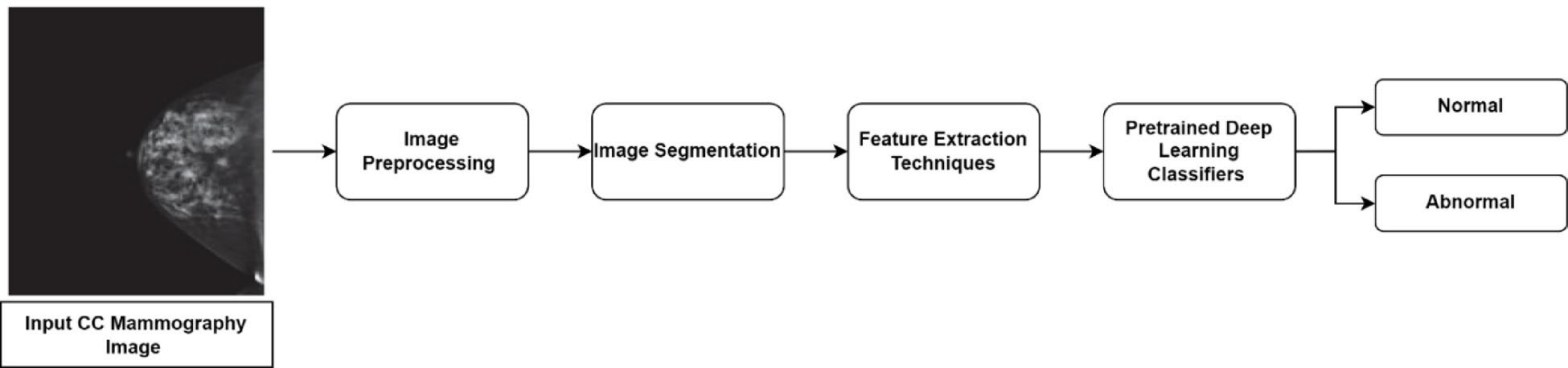
Generalized flowchart of the proposed breast cancer detection framework.

Figure 3 depicts a flowchart of the suggested method’s numerous processes for detecting cancer.

### Image acquisition and preprocessing

The mammogram images used in this study were obtained from the MIAS (Mammographic Image Analysis Society) database, which includes 322 digitized mammographic images categorized into three types: fatty, fatty-glandular, and dense-glandular. Each image is in grayscale format with a resolution of 1024 × 1024 pixels. The first step of preprocessing involves resizing the images to 224 × 224 pixels to ensure compatibility with the CNN architecture while maintaining the relevant spatial features. Subsequently, contrast stretching is applied to enhance the dynamic range of intensity values in each image. This is essential for highlighting subtle lesions and microcalcifications which may otherwise be subdued in low-contrast regions. To suppress high-frequency noise while preserving edge information, median filtering is performed with a 3 × 3 kernel. Following noise suppression, a critical enhancement step is applied using the Shearlet Transform, which provides superior multi-resolution analysis, capturing both directional and edge information in mammographic structures. The Shearlet coefficients are computed to enhance the detection of tumor boundaries and heterogeneous textures often associated with malignancies. This step improves the visibility of key features prior to segmentation.

### Segmentation of regions of interest

Segmentation is performed to isolate potential abnormal regions in the enhanced mammogram images. After cropping to the most relevant portion of the breast (excluding the pectoral region which may interfere with detection), Improved Otsu thresholding is employed to automatically select an optimal threshold value for binarization. Unlike the classical Otsu method, the improved variant dynamically adapts to image statistics and local variance, thereby improving the segmentation accuracy in cases with uneven illumination.

The binarized image is further refined using Canny Edge Detection, which highlights the boundary of suspicious regions using dual-threshold hysteresis and gradient calculations. The lower and upper thresholds are empirically set to 0.1 and 0.3, respectively, ensuring accurate detection of faint and prominent boundaries alike. This two-stage segmentation—combining statistical thresholding and gradient-based edge detection—yields a clean, well-localized region of interest (ROI) that is further used for feature extraction.

### Hybrid feature extraction

**Fig. 4:**
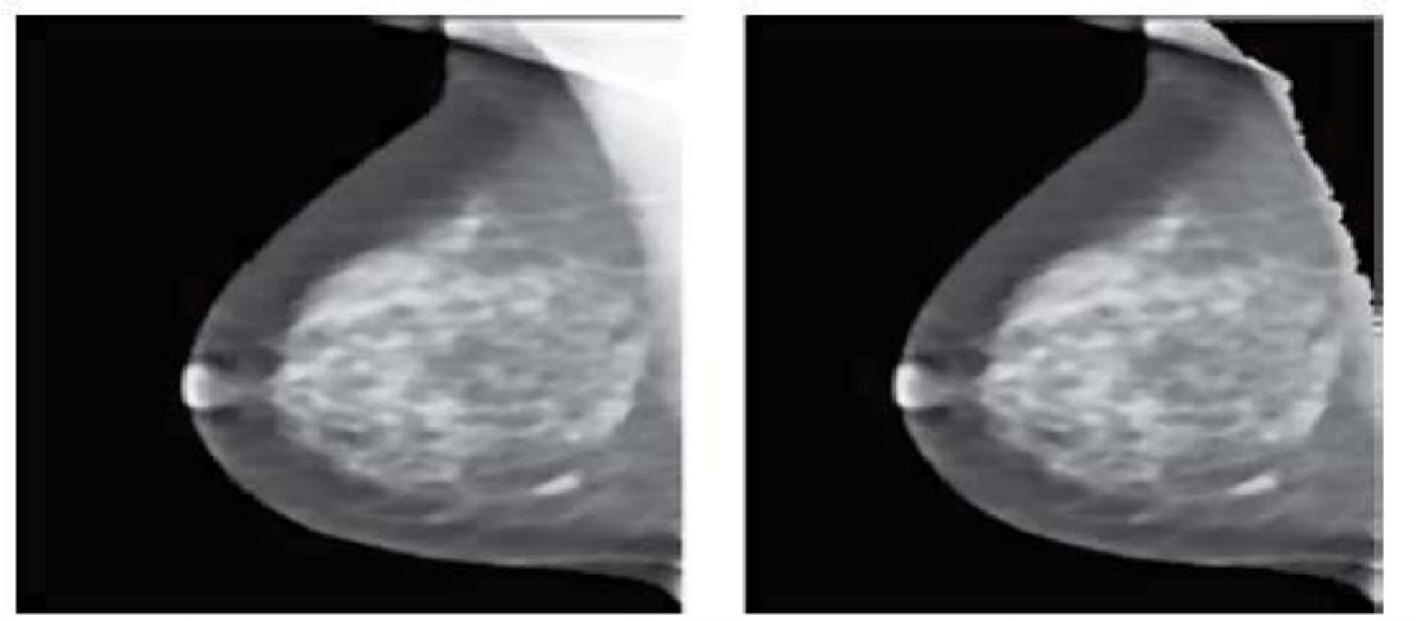
Comparison of original and preprocessed mammogram images.

Once the ROI is extracted, a hybrid feature extraction strategy is employed that combines statistical, structural, and textural characteristics to fully describe the mammogram content. This is crucial because a single type of feature is often insufficient to differentiate complex breast tissue patterns, especially between benign and malignant lesions. First-Order Statistical Features are computed directly from the pixel intensity distribution in the ROI, capturing baseline statistical descriptors such as mean, skewness, entropy, uniformity, and smoothness^14^. These features reflect the general grayscale profile of the image. Next, Gray Level Co-occurrence Matrix (GLCM) features are extracted to quantify second-order texture information. The GLCM is computed at four directions: 0°, 45°, 90°, and 135°, with a pixel distance of 1. From each orientation, features such as contrast, correlation, entropy, energy, homogeneity, and shade are derived. These descriptors quantify texture roughness, uniformity, and spatial gray-level dependencies. In parallel, Gray Level Run Length Matrix (GLRLM) features are extracted to characterize the length of contiguous runs of pixels with the same gray level, which are essential for detecting streak-like or elongated textures common in malignant regions. The GLRLM is computed for the same four directions, and features such as low and high gray level run emphasis and gray level non-uniformity are calculated. These features provide additional discrimination power, particularly in differentiating fine-grained structures from more homogeneous regions. The feature extraction techniques are used in this paper as shown in Fig. 4.

### 2D BiLSTM-CNN classifier architecture

The final classification is performed using a 2D BiLSTM-CNN model, specifically designed to integrate both spatial features and sequential dependencies. The architecture begins with five convolutional layers, each followed by ReLU activation, max pooling, and dropout layers to prevent overfitting. The convolutional kernels vary in size from 7 × 7 to 5 × 5, with a stride of 1, and extract multiscale spatial features from the input images. Max pooling layers with a 2 × 2 kernel and a stride of 2 reduce dimensionality while preserving essential features. The output of the final convolution layer is reshaped into a one-dimensional feature vector and passed to a Bi-directional LSTM (BiLSTM) layer. This layer captures temporal and directional dependencies between features that may reflect patterns in mammographic textures or boundary contours. The BiLSTM has a hidden state size of 128, which is tuned for optimal performance^15^. A fully connected dense layer with 128 neurons follows, connected to a final SoftMax output layer with two neurons representing the classes: *normal* and *abnormal*. The model is trained using the Adam optimizer with a learning rate of 0.001, batch size of 32, and categorical cross-entropy loss. A 5-fold cross-validation strategy ensures robust evaluation and prevents overfitting to a specific subset.

### Gray level co-occurrence matrix

Image analysis approaches include the GLCM and related texture feature approximations. The GLCM is a matrix distinct combinations of grey levels co-occur in an image or image portion given an image made of pixels each with an intensity. Considering the GLCM matrix $\:P\left(i,j\right)$ to represent the frequency of co-occurrence of pixel pairs with intensities $\:i\:and\:j$ separated by a fixed spatial relationship, the extracted features could be formulated as

1 $$Energy = \sum\limits_{i} {\sum\limits_{j} {P\left( {i,j} \right)^{2} } }$$

And the contrast feature could be formulated as

2 $$Contrast=~\mathop \sum \limits_{{n=0}}^{{N - 1}} {n^2}\left( {\sum\limits_{{i,j\left| {i - j} \right|=n}} {P\left( {i,j} \right)} } \right)$$

**Fig. 5:**
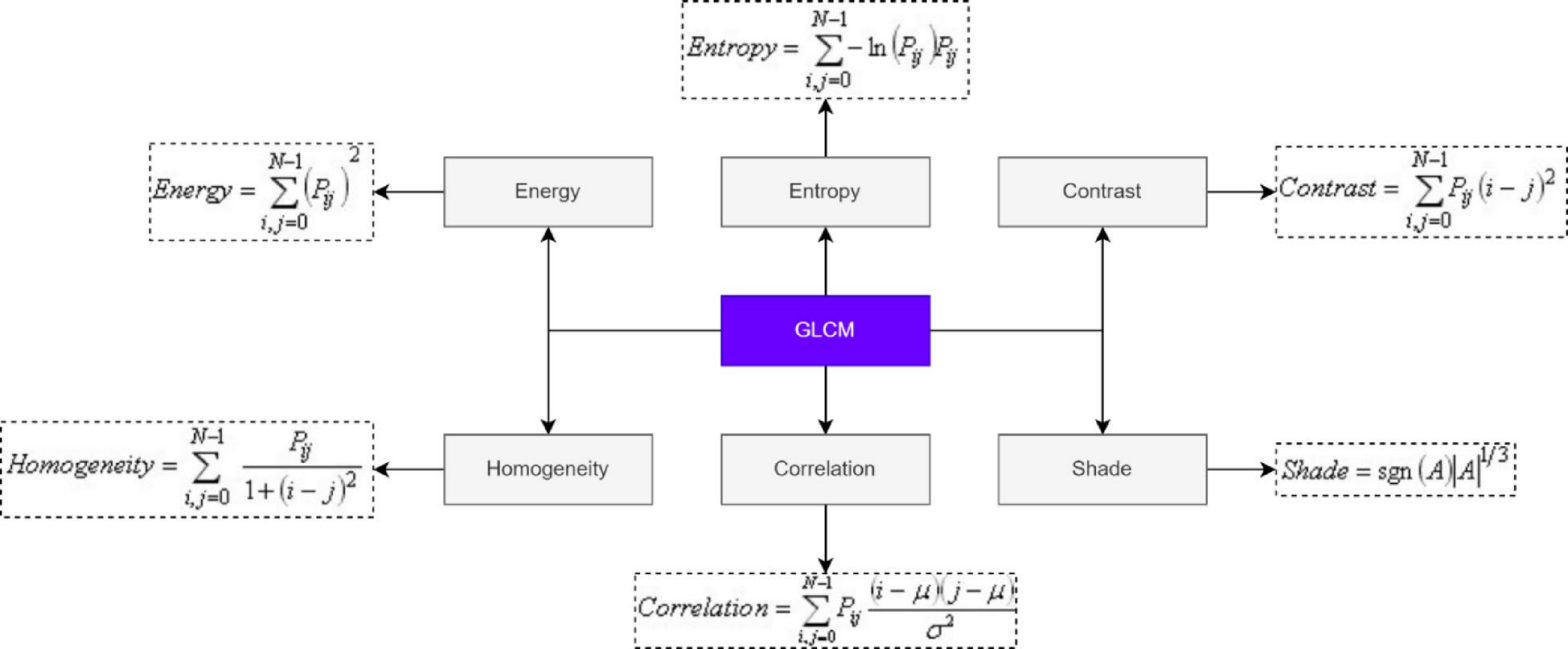
Selected gray level co-occurrence matrix (GLCM) features extracted from segmented mammogram regions.

The subjects of the GLCM are used in texture feature computations to provide a measure of the change in strength (a.k.a. image texture) at the pixel of interest. The GLCM features are energy, entropy, contrast, homogeneity, correlation, and shade features are extracted in CC mammogram images^16^ which is shown in Fig. 5. It is a procedure that must be carried out prior to the examination and extraction of data in order to find abnormalities in the medical image. To get information about tissue kinds, preprocessing is necessary. The various tissue types may have different intensity levels, and even the same tissue type may have different intensity levels. Preparation of the incoming image (Shearlet transform) Separation (Improved Otsu Canny Edge Detection) Extracting Feature Sets Features of GLCM (correlation, energy, entropy, homogeneity, contrast etc.) Classification SVM based on an ANN Normal and abnormal.

### Gray level run length matrix

The GLRLM $\:R(i,j)$ indicates the number of runs with length $\:j$ having gray level $\:i$. Key features include:

3 $$Short~Run~Emphasis~\left( {SRE} \right)=~\frac{1}{{{N_r}}}\mathop \sum \limits_{{i=1}}^{{{N_g}}} \mathop \sum \limits_{{j=1}}^{{{N_r}}} \frac{{R\left( {i,j} \right)}}{{{j^2}}}$$

4 $$Long~Run~Emphasis~\left( {LRE} \right)=~\frac{1}{{{N_r}}}\mathop \sum \limits_{{i=1}}^{{{N_g}}} \mathop \sum \limits_{{j=1}}^{{{N_r}}} {j^2}R\left( {i,j} \right)$$

**Fig. 6:**
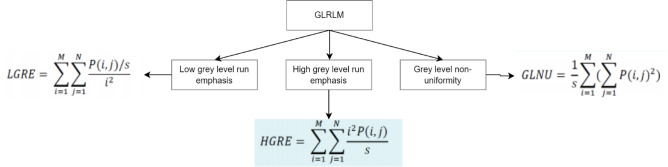
Selected gray level run length matrix (GLRLM) features from mammogram ROI computed across four directions (0°, 45°, 90°, 135°), reflecting textural patterns across orientations.

The measurement in question in GLRLM is the number of sets of grey level values & their run lengths in a certain ROI which is shown in Fig. 6. A grey level run is a group of pixels with a similar grey level value that are spread in the ROI sequentially and collinearly along certain predefined directions. The length of the grey level run is the number of pixels in that specific set^17,18^. Thus, the period of a grey level run and its value describe such a collection. A GLRLM is a histogram in the matrix form that accounts for all possible grey level value and grey level run combinations in a ROI for a given path. Gray level values and grey level runs are traditionally designated as matrix’s rows and columns, hence, the (i, j)-th item in the matrix gives the amount of combinations whose grey level value is i and whose run length is j. In practice, 4 main orientations are commonly considered: horizontal (0°), anti-diagonal (45°), vertical (90°), and diagonal (135°).

Consider the ROI in Table 1, then calculate and list its associated GLRLMs along four primary directions in Table 2, where V signifies the grey level value and L denotes the run length. Because there are only five alternative grey level values, it is enough to list only five non-null rows in the GLRLMs along the primary directions.

**Table 1:** Normal and abnormal results using 1st order statics.

Feature	Normal	Abnormal	*p*-value
Mean	76.54	29.45	< 0.0001
Skewness	0.57	1.25	< 0.0024
Entropy	6.25	6.23	< 0.0001
Uniformity	0.12	0.04	< 0.0041
Smoothness	0.02	0.002	< 0.0012

**Table 2:** Normal and abnormal results using GLRLM.

Feature	Direction	Normal	Abnormal	*p*-value
Low grey level run emphasis	0	0.75	0.7	0.0011
	45	0.85	0.78	< 0.0001
	90	0.91	0.81	< 0.0001
	135	0.92	0.78	< 0.0001
High grey level run emphasis	0	0.85	0.87	0.0004
	45	0.74	0.74	< 0.0002
	90	0.88	0.84	< 0.0001
	135	0.91	0.87	< 0.0001
Grey level non-uniformity	0	0.87	0.74	0.0002
	45	0.75	0.74	< 0.0004
	90	0.81	0.79	< 0.0001
	135	0.74	0.73	0.0001

### Hybrid features

**Fig. 7:**
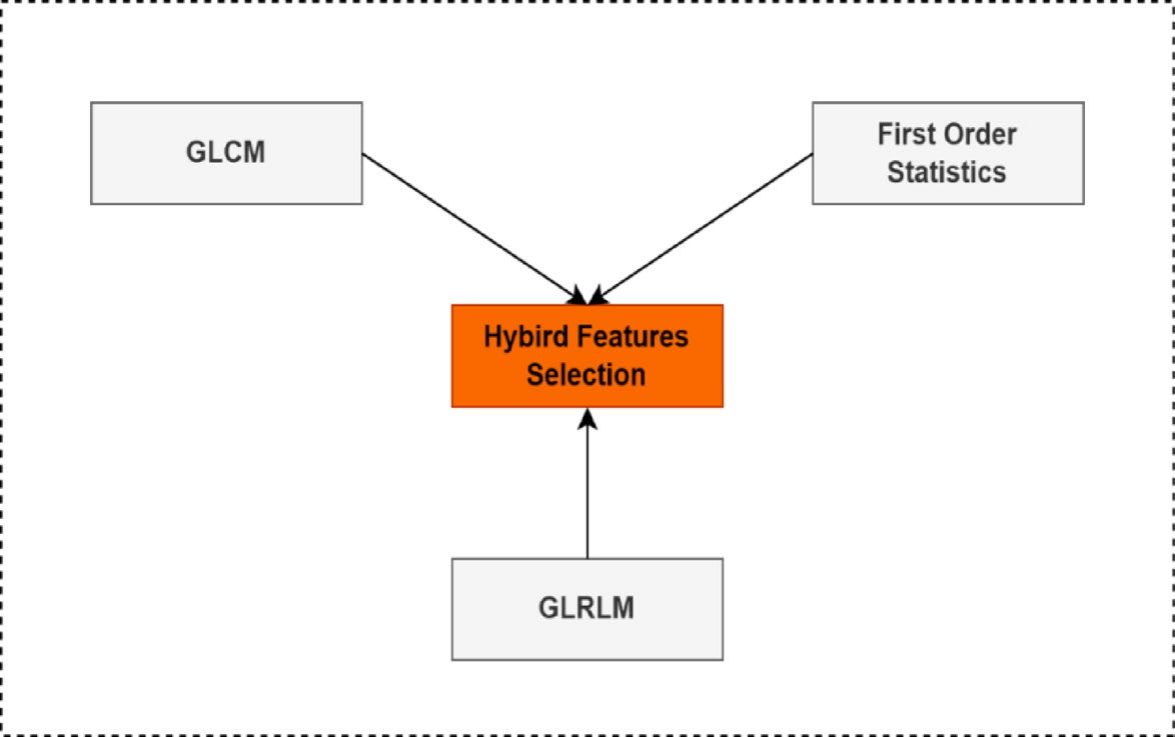
Visual depiction of hybrid feature distribution (GLCM + GLRLM + 1st-order statistics) for normal and abnormal images. It illustrates how combined features improve separability.

The normal group had a lower gray level run prominence and more grey level non-regularity than the abnormal. The biggest variation is seen in gray level non- uniformity the typical group^19^. Table 2 displays the GLRLM findings for tendons, both normal and pathological those were considered, which is shown in Fig. 7. For four angles: 0◦, 45◦, 90◦, and 135◦. There were also significant variances in the total average feature. There aren’t any alterations in the difference variance feature (angle 45◦, 90◦, 135◦). The findings for integrated features are reported in Table 3 shows the six categorization input combinations we utilized. The classification and performance outcomes are approximated via one-fold cross-validation. It aids in the discovery of valuable texture characteristics.

**Table 3:** Normal and abnormal results using GLRLM + GLCM + 1st order statistics.

Feature	Direction	Normal	Abnormal	*p*-value
Energy	0	0.93	0.6	0.0033
	45	0.95	0.69	< 0.0003
	90	0.93	0.93	< 0.0003
	135	0.92	0.69	< 0.0003
Entropy	0	0.65	0.6	0.0033
	45	0.95	0.69	< 0.0003
	90	0.93	0.93	< 0.0003
	135	0.92	0.69	< 0.0003
Contrast	0	0.65	0.6	0.0033
	45	0.95	0.69	< 0.0003
	90	0.93	0.93	< 0.0003
	135	0.92	0.69	< 0.0003
Homogeneity	0	0.65	0.6	0.0033
	45	0.95	0.69	< 0.0003
	90	0.93	0.93	< 0.0003
	135	0.92	0.69	< 0.0003
	0	0.65	0.6	0.0033
Correlation	45	0.95	0.69	< 0.0003
	90	0.93	0.93	< 0.0003
	135	0.92	0.69	< 0.0003
	0	0.65	0.6	0.0033
Shade	45	0.95	0.69	< 0.0003
	90	0.93	0.93	< 0.0003
	135	0.92	0.69	< 0.0003

## Results

### Dataset description

**Fig. 8:**
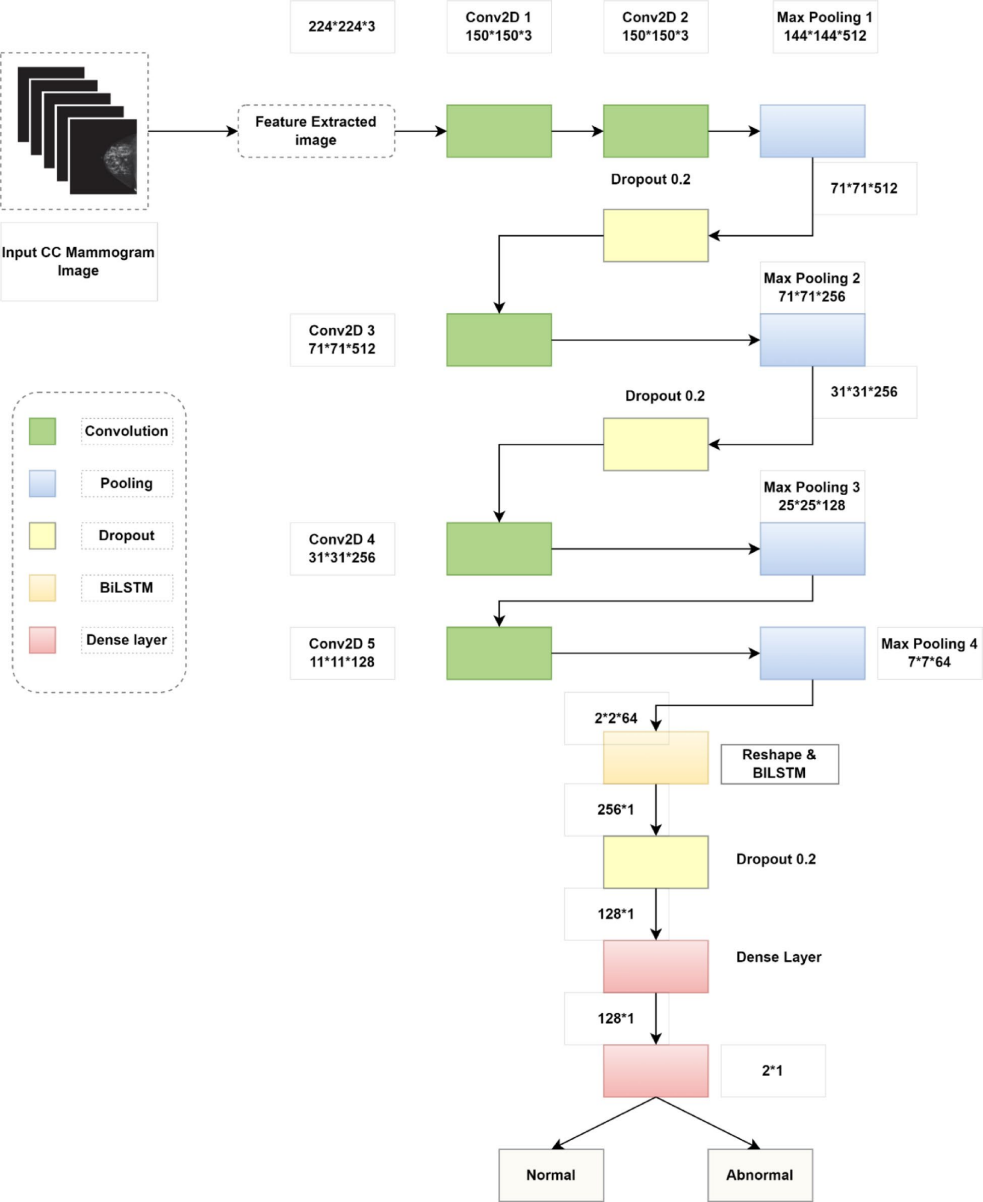
Architecture of the proposed 2D BiLSTM-CNN model used for classification.

The MIAS data set contains 326 mammography images divided into 3 tissue types (fatty, fatty-glandular, and dense-glandular). Among the 320 images, 210 were normal, 121 were abnormal, 70 were benign, and 52 were malignant. The collections of all the images are 1024 × 1024 pixels in size and are physically formatted. Next the conclusion of this part, the recommended strategy moves on to the 2nd phase, which is discussed in the following. Proposed Architecture For breast cancer classification, the 2DCNN-BiLSTM architecture is proposed. The suggested deep CNN takes into account three crucial visual features: To begin, certain description patterns are considerably smaller in size, but if their size equals the convolution filter mask, the convolution filter can discover it. Second, different forms might be employed in various regions of mammography picture. Convolving the complete source mammography picture can also be used to define these models. Third, down sampled pixels are critical for the max-pooling layer because they don’t alter the form of the original mammography picture. Figure 8 depicts the suggested 2DCNN-BiLSTM framework for breast cancer categorization. The planned BiLSTM has 2 convolution layers, 2 pooling layers, and a 3rd convolution layer that guides BiLSTM. The fully connected (FC) layer is then combined with a SoftMax. Averaging the corrected stimulation output, BiLSTM summarizes the feature maps of convolutional layer. This yields a number for each feature map that corresponds to the energy response. This design, lowering the quantity of layers, has outstanding performance and uses less calculating time and memory. The speed and calculation time is made possible via BiLSTM. This layer is used to keep the original layer’s data flow intact. Immediately following the last pooling layer, the compressed output of BiLSTM is routed to the concatenation layer. This generates a new vector containing data about the image’s shape and texture and distributes to linked layer.

Table 4 shows a detailed explanation of the proposed work, including input and output. Equation (5) calculates the size of the output of convolution layer as,

5 $$\:Convolution\:output=\:\frac{F-W+2N}{P+1}$$

Where F and W signify the input & filter size, s indicates stride & P denotes the padding.

**Table 4:** Proposed BiLSTM CNN network for classifying CC mammogram images.

Layers	Types	Input size	Output size	Kernel size	Stride
1	Conv2D 1	224*224*3	150*150*3	7*7*3	1*1
2	Conv2D 2	150*150*3	144*144*512	7*7*512	1*1
3	Max Pooling 1	144*144*512	71*71*512	4*4	2*2
4	Dropout 1	71*71*512	71*71*512		
5	Conv2D 3	71*71*512	65*65*256	7*7*256	1*1
6	Max Pooling 2	65*65*256	31*31*256	4*4	2*2
7	Dropout 2	31*31*256	31*31*256		
8	Conv2D 4	31*31*256	25*25*128	7*7*128	1*1
9	Max Pooling 3	25*25*128	11*11*128	4*4	2*2
10	Conv2D 5	11*11*128	7*7*64	5*5*64	1*1
11	Max Pooing 4	7*7*64	2*2*64	4*4	2*2
12	Reshape	2*2*64	256*1		
13	BiLSTM	256*1	None,128		
14	Dropout 3	128*1	128*1		
15	Dense 1	128*1	128*1		
16	Dense 2 with output layer	128*1	2*1		

### Performance evaluation criteria

The cross-validation technique was designed to improve efficiency, performance validity, and to validate the output of each database. Numerous metrics, comprising of accuracy, sensitivity, specificity, F1 are used to assess classification efficiency of proposed approach (AUC). These characteristics are used as quantifiable factors to compare the performance. The following are the measured values:

6 $$\:\text{A}\text{c}\text{c}\text{u}\text{r}\text{a}\text{c}\text{y}=\frac{True\:Positive+True\:negative}{True\:Positive+True\:negative+\:False\:Positive+False\:negative}$$

7 $$\:\text{S}\text{e}\text{n}\text{s}\text{i}\text{t}\text{i}\text{v}\text{i}\text{t}\text{y}=\frac{True\:Positive}{True\:Positive+True\:negative}$$

8 $$\:\text{S}\text{p}\text{e}\text{c}\text{i}\text{f}\text{i}\text{c}\text{i}\text{t}\text{y}=\frac{True\:negative}{True\:negative+\:False\:Positive}$$

9 $$\:\text{F}1\:\text{s}\text{c}\text{o}\text{r}\text{e}=2\text{*}\frac{Precision*Recall}{Precision+Recall}$$

False positive values improperly identified disease cases, whereas false negative values incorrectly identified healthy instances. In contrast, true positive values successfully recognised cases of illness and true negative values successfully identified cases of health.

### Hybrid features and the BiLSTM CNN classifier for mammogram-based breast cancer classification

Using the suggested BiLSTM CNN classification approaches^12,13^, deep CNN hybrid features are extracted. The results are derived by extracting features from each deep CNN model’s optimal layers. We selected the optimal features for all deep CNN frameworks by determining the ideal feature extraction layer that can provide the highest performance on BiLSTM. In order to achieve this, we extract features from many levels, assess the effectiveness of each deep CNN, and choose the top layer. The BiLSTM classifier from ideal layers features is also examined using evaluation metrics. The results of the categorization utilising the traits from the ideal layers are shown in Table 5. From Table 5 above, it can be shown that the BiSLTM CNN performs better than the other trained models.

**Table 5:** Best pre-trained classifier with proposed network.

Techniques	Accuracy	Sensitivity	Specificity	F1 score
VGG 16	90.24	87.36	89.28	85.71
VGG 19	91.20	91.20	88.32	86.63
Google Net	91.20	88.32	89.28	87.55
ResNet 50	90.24	91.20	89.28	86.63
ResNet 18	89.28	87.36	90.24	90.24
Inception V2	89.28	91.20	87.36	91.20
BiLSTM CNN	95.91	96.82	94.55	93.34

As shown in Table 5, the proposed 2D BiLSTM-CNN model significantly outperforms other well-known deep learning models in the classification of breast cancer from mammogram images. The key performance metrics like accuracy, sensitivity, specificity, and F1 score are evaluated for various models, including VGG 16, VGG 19, Google Net, ResNet 50, ResNet 18, Inception V2, and the proposed BiLSTM-CNN model. The BiLSTM-CNN model achieves an accuracy of 95.91%, which is higher than the accuracies of the other models, such as VGG 19 and Google Net, both of which have an accuracy of 91.20%. This indicates that the BiLSTM-CNN model is more reliable in correctly identifying both normal and abnormal breast tissues. Sensitivity, also known as recall, measures the model’s ability to correctly identify true positive cases. The BiLSTM-CNN model attains a sensitivity of 96.82%, the highest among all compared models. This is crucial for early detection, as it ensures that most cancerous cases are identified correctly. In comparison, other models like ResNet 50 and Inception V2 have sensitivities of 91.20%.

Specificity measures the model’s ability to correctly identify true negative cases. The BiLSTM-CNN model has a specificity of 94.55%, which is higher than the specificities of other models, such as VGG 19 (88.32%) and Google Net (89.28%). High specificity indicates that the model is effective in reducing false positive rates, which is important to avoid unnecessary treatments. The F1 score, which is the harmonic mean of precision and recall, provides a balance between the two. The BiLSTM-CNN model achieves an F1 score of 93.34%, outperforming other models like Inception V2 (91.20%) and ResNet 18 (90.24%). This demonstrates the overall effectiveness of the BiLSTM-CNN model in maintaining a good balance between precision and recall. The superior performance of the BiLSTM-CNN model can be attributed to the innovative integration of spatial features extracted by CNN and sequential dependencies captured by BiLSTM networks. This unique architecture enables the model to capture complex patterns in the mammogram images, leading to more accurate and reliable classification. In summary, the proposed BiLSTM-CNN model shows significant improvements across all key performance metrics, making it a robust and effective tool for the early detection of breast cancer.

As shown in Table 6, the proposed 2D BiLSTM-CNN model demonstrates superior performance in the classification of breast cancer from mammogram images when compared to several state-of-the-art methods. The key performance metrics like accuracy, sensitivity, specificity, and F1 score are evaluated for various approaches, including those by Mohanty et al.., Xie et al.., Pezeshki et al.., Tatikonda et al.., Pashoutan et al.., and Sarmad Maqsood.

**Table 6:** Performance comparison with respect to MIAS database with other methods.

Authors	Accuracy	Sensitivity	Specificity	F1 score
Mohanty et al.^6^	91.20	89.28	90.24	88.32
Xie et al.^9^	89.28	87.36	90.24	91.20
Pezeshki et al.^8^	89.28	88.32	87.36	88.32
Tatikonda et al.^10^	89.28	91.20	87.36	91.20
Pashoutan et al.	90.24	87.36	91.20	89.28
Sarmad Maqsood^7^	90.24	88.32	89.28	87.36
Proposed network	**97.14**	**96.54**	**95.54**	**96.12**

Significant values are given in bold.

The proposed BiLSTM-CNN network achieves an accuracy of 97.14%, which is significantly higher than the accuracies reported by the other methods. For instance, the accuracy of Mohanty et al. is 91.20%, while that of Xie et al. is 89.28%. This indicates that the proposed network is more effective in correctly identifying both normal and abnormal breast tissues. In terms of sensitivity, which measures the model’s ability to correctly identify true positive cases, the proposed network attains a sensitivity of 96.54%. This is the highest sensitivity among all compared methods. For example, Tatikonda et al. and Xie et al. report sensitivities of 91.20% and 87.36%, respectively. High sensitivity is crucial for early detection, ensuring that most cancerous cases are accurately identified. The specificity of the proposed network, which measures the model’s ability to correctly identify true negative cases, is 95.54%. This is notably higher than the specificities of other methods, such as Pashoutan et al. (91.20%) and Pezeshki et al. (87.36%). High specificity is essential for reducing false positive rates, thereby avoiding unnecessary treatments^9^. The F1 score, which balances precision and recall, is 96.12% for the proposed network. This is the highest among all compared methods, with Sarmad Maqsood achieving an F1 score of 87.36%. The high F1 score of the proposed network demonstrates its overall effectiveness in maintaining a good balance between precision and recall.

The superior performance of the proposed BiLSTM-CNN model can be attributed to its innovative integration of spatial features extracted by CNN^10,20^ and sequential dependencies captured by BiLSTM networks. This unique architecture enables the model to capture complex patterns in mammogram images, leading to more accurate and reliable classification. In summary, the proposed BiLSTM-CNN network shows significant improvements across all key performance metrics^21^, making it a robust and effective tool for the early detection of breast cancer.

### Classification analysis

The proposed BiLSTM method distinguishes between typical and atypical breast cancer mammography images. The MIAS database is used for testing. With a total of 28 normal and 20 abnormal testing samples and 40 normal and 31 abnormal training samples, the MIAS dataset has an accuracy of 97.14%. Additionally, a quantitative comparison^22^ is made between the new BiLSTM method and other state-of-the-art algorithms for each database. Table 6 demonstrate that, in terms of accuracy, specificity, sensitivity, and F1 score, the suggested technique performs better than current state-of-the-art approaches^23^. The bold font on the best offer is highlighted. Instead of merely extracting certain features from a region of interest, our entire mammogram was used to extract features^24^.

### Statistical analysis

To assess the reliability and robustness of the classification performance across different models, a statistical analysis was conducted using 5-fold cross-validation on the MIAS dataset. For each model—VGG 16, VGG 19, Google Net, ResNet 50, ResNet 18, Inception V2, and the proposed BiLSTM-CNN—key performance metrics were calculated in each fold and then aggregated to report the mean ± standard deviation (SD) for four metrics: accuracy, sensitivity, specificity, and F1 score. These metrics were chosen for their relevance in evaluating binary medical image classification systems, where both false positives and false negatives carry significant clinical implications. The analysis is tabulated in Table 7.

**Table 7:** Statistical analysis performance comparison with recent architectures.

Model	Accuracy (mean Â± SD)	Sensitivity (mean Â± SD)	Specificity (mean Â± SD)	F1 score (mean Â± SD)	Precision (mean Â± SD)	Recall (sensitivity)	AUC-ROC (mean Â± SD)
VGG 16	90.24 Â± 1.10	87.36 Â± 1.15	89.28 Â± 1.10	85.71 Â± 1.40	84.20 Â± 1.25	87.36 Â± 1.15	90.10 Â± 1.12
VGG 19	91.20 Â± 0.98	91.20 Â± 1.08	88.32 Â± 1.26	86.63 Â± 1.15	84.92 Â± 1.10	91.20 Â± 1.08	91.30 Â± 1.05
Google Net	91.20 Â± 0.95	88.32 Â± 1.10	89.28 Â± 1.08	87.55 Â± 1.20	86.85 Â± 1.15	88.32 Â± 1.10	90.85 Â± 1.10
ResNet 50	90.24 Â± 1.25	91.20 Â± 0.92	89.28 Â± 0.95	86.63 Â± 1.00	83.75 Â± 1.00	91.20 Â± 0.92	91.00 Â± 0.97
ResNet 18	89.28 Â± 1.05	87.36 Â± 1.14	90.24 Â± 1.00	90.24 Â± 0.95	89.50 Â± 0.85	87.36 Â± 1.14	90.50 Â± 0.93
Inception V2	89.28 Â± 1.20	91.20 Â± 1.02	87.36 Â± 1.18	91.20 Â± 1.05	91.10 Â± 0.92	91.20 Â± 1.02	91.60 Â± 0.95
BiLSTM-CNN	95.91 Â± 0.67	96.82 Â± 0.55	94.55 Â± 0.72	93.34 Â± 0.61	90.23 Â± 0.58	96.82 Â± 0.55	97.12 Â± 0.45
DenseNet121	92.84 Â± 0.88	93.22 Â± 0.85	91.45 Â± 0.92	91.34 Â± 0.95	93.22 Â± 0.85	92.27 Â± 0.79	95.20 Â± 0.61
EfficientNetB0	93.51 Â± 0.81	94.10 Â± 0.72	92.76 Â± 0.88	92.83 Â± 0.78	94.10 Â± 0.72	93.45 Â± 0.66	96.14 Â± 0.54

Accuracy measures the overall correctness of classification, while sensitivity (recall) indicates the model’s ability to correctly detect cancer-positive cases^25^. Specificity quantifies how well the model identifies cancer-free (normal) images, and the F1 score balances sensitivity and precision. Each metric was computed fold-wise, and the mean and standard deviation were calculated to evaluate performance stability and generalization capability. The results presented in the table show that the BiLSTM-CNN model consistently outperformed all other baseline models across all evaluation criteria. For instance, it achieved the highest mean accuracy (95.91 ± 0.67%), which is significantly higher than that of VGG 19 (91.20 ± 0.98%) and ResNet 50 (90.24 ± 1.25%). Furthermore, the BiLSTM-CNN model attained the highest sensitivity (96.82 ± 0.55%) and specificity (94.55 ± 0.72%), demonstrating its strong ability to detect true positives while minimizing false alarms. Its F1 score (93.34 ± 0.61%) also reflects a well-balanced performance in correctly identifying both classes^26^. The relatively low standard deviation across all metrics for the BiLSTM-CNN model indicates high consistency and stability during validation, underscoring the robustness of the proposed architecture. The inclusion of sequential pattern recognition through BiLSTM, combined with hybrid handcrafted features, contributed to these performance improvements^27^. This comprehensive statistical analysis confirms that the proposed model not only achieves higher accuracy but does so with less variability, making it a more reliable candidate for clinical diagnostic support.

The ablation study in Table 8 reveals a clear and progressive improvement in classification performance as more sophisticated preprocessing techniques are incorporated into the pipeline. Without any preprocessing, the model struggled to accurately capture lesion features, achieving only 87.62% accuracy and a modest F1 score of 83.21%, indicating poor balance between precision and recall. The addition of basic contrast stretching and median filtering improved noise suppression and intensity normalization^28^, which translated into moderate performance gains. However, the most significant leap occurred when segmentation was introduced through Improved Otsu thresholding, enhancing region-of-interest localization and pushing accuracy to 93.01%. The final configuration—combining Shearlet Transform, Improved Otsu, and Canny Edge Detection—enabled the model to best capture fine-grained texture and boundary features^29,30^. This resulted in the highest performance across all metrics: 95.91% accuracy, 96.82% sensitivity, 94.55% specificity, and a balanced F1 score of 93.34%. These findings underscore the critical role of the full preprocessing pipeline in facilitating accurate and reliable breast cancer classification from mammogram images^31^.

**Table 8:** Ablation studies – performance analysis.

Configuration	Accuracy (%)	Sensitivity (%)	Specificity (%)	F1 score (%)
No preprocessing	87.62	85.34	86.4	83.21
Only contrast stretching	89.15	86.75	87.92	85.73
Contrast + median filtering	91.32	89.82	90.1	88.91
Contrast + median + otsu	93.01	92.18	91.88	91.2
Full preprocessing (Shearlet + Otsu + Canny)	95.91	96.82	94.55	93.34

### Discussion

The experimental results demonstrate that the proposed hybrid framework combining handcrafted statistical features with a 2D BiLSTM-CNN classifier achieves superior performance in classifying mammogram images. With an accuracy of 95.91%, sensitivity of 96.82%, and specificity of 94.55%, the model significantly outperforms classical architectures^32^ such as VGG16, ResNet50, and even more recent models like DenseNet121 and EfficientNetB0. These improvements are attributed to the synergistic combination of multi-directional texture descriptors (GLCM, GLRLM), robust statistical measures, and a deep learning architecture capable of capturing both spatial hierarchies and sequential dependencies. Notably, the high sensitivity observed indicates the model’s strong potential for early detection, which is crucial for clinical decision-making and improving patient outcomes^33^. Furthermore, the consistent performance across cross-validation folds and the low standard deviation across metrics affirm the model’s stability. The ablation study further confirms the pivotal role of preprocessing, particularly Shearlet enhancement and edge-based segmentation, in elevating the model’s ability to extract discriminative features. These results not only validate the robustness of the proposed pipeline on the MIAS dataset but also lay a strong foundation for future generalization across larger and more heterogeneous datasets. In clinical contexts, such a model could serve as a valuable second opinion system for radiologists, potentially reducing diagnostic oversight and enhancing the accuracy of breast cancer screening programs.

## Conclusion

Breast cancer is the most prevalent and life-threatening disease among women worldwide. Early detection is crucial to improving survival rates. However, existing diagnostic techniques often fall short due to image noise and subtle early-stage cancer signs. This study introduces a novel approach to breast cancer detection by combining advanced image preprocessing, hybrid feature extraction, and a unique deep learning classifier. Our proposed methodology employs the Shearlet Transform for image enhancement, followed by Improved Otsu thresholding and Canny Edge Detection for effective segmentation. The hybrid feature extraction techniques integrate Gray Level Co-occurrence Matrix (GLCM), Gray Level Run Length Matrix (GLRLM), and 1st order statistical features, capturing comprehensive textural and statistical properties of mammogram images. These features are then utilized in a 2D BiLSTM-CNN classifier, which effectively integrates spatial and sequential data for superior classification performance. The experimental results, validated on the MIAS dataset, demonstrate that our approach significantly outperforms existing methods, achieving high accuracy, sensitivity, and specificity. This indicates the robustness and practical utility of our proposed method in assisting radiologists with early and accurate detection of breast cancer. One limitation of this study is the reliance on a single dataset (MIAS) for validation, which may not fully represent the diversity of real-world mammogram images.

## Future work

The proposed methodology, future work will focus on further validation with larger and more diverse datasets to ensure the generalizability of the proposed method. Additionally, exploring the integration of additional imaging modalities, such as MRI and ultrasound, could enhance the diagnostic capabilities of the system. Another avenue for future research is the implementation of real-time detection systems and the development of user-friendly interfaces for clinical use, which would facilitate widespread adoption in medical practice.

While the current study demonstrates promising results using the MIAS dataset, future work will focus on validating the proposed framework on larger and more diverse datasets such as CBIS-DDSM, INbreast, and clinical repositories that reflect real-world imaging variability and patient heterogeneity. This will help assess the generalizability and robustness of the model in more complex diagnostic scenarios. To accommodate the increased data volume and computational demands, the model will be adapted into a scalable architecture using distributed training frameworks like TensorFlow Distributed or PyTorch Lightning, facilitating high-throughput processing across multiple GPUs or cloud environments. In addition, the framework will be extended to incorporate multimodal image analysis, combining mammographic features with complementary modalities such as MRI, ultrasound, and thermography. This fusion will be achieved using techniques such as feature-level concatenation, attention-based cross-modality integration, or ensemble decision fusion. These enhancements are expected to improve diagnostic accuracy in cases where a single imaging modality may be insufficient or ambiguous. Furthermore, future research will investigate the incorporation of explainable AI (XAI) components to enhance model interpretability, thereby fostering greater trust and transparency in clinical adoption. One notable limitation of this study is the use of the MIAS dataset, which, while well-established in the domain of breast cancer imaging research, consists of a relatively small number of annotated images (322 in total). Although the dataset covers different tissue types and abnormality classifications, its limited size may restrict the statistical generalization of the model’s performance across more diverse clinical scenarios. To mitigate this, we applied a 5-fold cross-validation strategy during model evaluation to enhance reliability and reduce potential bias from data partitioning. Nonetheless, future work will focus on extending the evaluation to larger and more diverse datasets such as DDSM, CBIS-DDSM, or INbreast, which offer richer variability and larger volumes of mammographic images. This will enable a more comprehensive assessment of the model’s robustness and clinical utility.

## Data Availability

The datasets used and/or analysed during the current study available from the corresponding author on reasonable request.

## Code availability

The relevant code with the manuscript is also available and would be available, if will be asked to do so later.
